# Capturing Value: How Health-System Specialty Pharmacies Define and Document Pharmacist Interventions

**DOI:** 10.3390/pharmacy13060172

**Published:** 2025-11-26

**Authors:** Autumn D. Zuckerman, Karen C. Thomas, Erica Diamantides, Shannan Takhar, Rushabh Shah, Kelsi Conant, Thom Platt, Christian Rhudy

**Affiliations:** 1 Vanderbilt Specialty Pharmacy, Vanderbilt Health, Nashville, TN 37211, USA; 2 Retzky College of Pharmacy, University of Illinois Chicago, Chicago, IL 60612, USA; 3 Tufts Medicine Pharmacy, Tufts Medicine, Boston, MA 02111, USA; 4 University of California San Francisco School of Pharmacy, University of California Davis Health, San Francisco, CA 94143, USA; 5 University of North Carolina Health Specialty and Home Delivery Pharmacy, University of North Carolina Health, Morrisville, NC 27560, USA; 6 West Virginia University Medicine Specialty Pharmacy, West Virginia University Medicine, Morgantown, WV 26505, USA; kelsi.conant@wvumedicine.org; 7 University of Kentucky HealthCare Specialty Pharmacy and Infusion Services, University of Kentucky HealthCare, Lexington, KY 40536, USA

**Keywords:** pharmacists, documentation, electronic health records, health-system pharmacy, interventions

## Abstract

Standardized pharmacist intervention practices and documentation among health-system specialty pharmacies could improve understanding of the pharmacists’ role and value in this setting. This study describes current health-system specialty pharmacies’ intervention practices. A survey developed by a volunteer committee subgroup was distributed to two health-system specialty pharmacy group email distribution lists. The survey evaluated the types of tasks considered to be clinical or non-clinical interventions; who could perform interventions; where and how they were documented; data elements included in documentation; and how intervention data were classified, used, reviewed, and shared with internal or external stakeholders. Twenty-four institutions responded to the survey. Tasks within medication management, adverse drug events/monitoring, and education domains were more commonly considered clinical interventions; tasks in the health maintenance and coordination of care domains were more frequently considered non-clinical interventions or not considered to be interventions. Interventions were completed by pharmacists (at 100% of sites) and were mostly documented in the electronic health record (92%). Intervention data were primarily collected to meet accreditation purposes (96%) or for quality auditing and review (88%). No respondents shared intervention data with patients. Results demonstrate areas of alignment and variance in intervention definition and documentation among health-system specialty pharmacies.

## 1. Introduction

The term “intervention” is commonly used throughout pharmacy literature, yet guidance on what to consider an intervention is limited. There are numerous examples of how pharmacists perform and document clinical interventions, resulting in positive patient outcomes across care settings [1,2,3,4,5,6,7,8]. Pharmacist interventions can often be tied to direct and indirect cost avoidance, most often avoiding costs for the patient and healthcare payor [9]. Determining what constitutes an intervention and assigning clinical and financial value to those interventions is needed to justify pharmacy resources and the pharmacist’s role in patient care.

Health-system specialty pharmacies (HSSPs) are integrated advanced practice models wherein pharmacists manage patients prescribed specialty medications from the health system across the continuum of care [10]. HSSPs are unique in that pharmacy services span from medication selection through medication access, initiation, monitoring, optimization, and ongoing coordination of care [11]. HSSP services are often provided to patients cared for by the health system regardless of their ability to receive dispensing services from the HSSP [11]. The HSSP model has shown several advantages, such as increased adherence and persistence, improved clinical outcomes, financial savings for patients, payers, and the healthcare system, enhanced quality of care, greater patient and provider satisfaction, and highly efficient specialty pharmacy services [12]. Almost all HSSPs have at least one specialty pharmacy accreditation (i.e., URAC, Accreditation for Healthcare Commission [ACHC], or The Joint Commission [TJC]) [13]. Accreditation requirements vary by the accrediting bodies, but all have components focusing on the documentation of clinical intervention(s) and the outcomes from those interventions.

Despite the benefit of the HSSP model, HSSPs continue to be excluded from payer and drug manufacturer networks, resulting in many HSSPs being able to dispense less than half of the specialty medication prescriptions generated at their health system [11]. To better demonstrate the value of the HSSP model to external stakeholders, HSSPs need to be able to clearly articulate pharmacist interventions and the resulting impact on clinical and financial outcomes. However, inter-institutional variability in intervention definitions hinders aggregation and interpretation of HSSP intervention data at scale to demonstrate these outcomes.

Because of their integration, HSSPs have valuable opportunities for targeted interventions by leveraging electronic health records (EHRs) to monitor laboratory results, imaging, medical history, and provider notes. Transparency into the EHR enables HSSPs to identify and address potential therapy-related issues, such as nonadherence, medication appropriateness, or adverse effects, and communicate these findings to the entire healthcare team. Though growing evidence demonstrates the value of clinical interventions supported by specialty pharmacists, there has been little evaluation of how these interventions can be documented, tracked, and shared with stakeholders. A survey of HSSPs administered by the American Society of Health-System Pharmacists (ASHP) showed that most HSSPs perform clinical interventions, though the types of interventions varied [11]. Additionally, URAC-accredited specialty pharmacies are required to track and document clinical interventions as part of their patient management programs [14]. However, there is little guidance on how specialty pharmacies should do this. The objective of this national cross-sectional survey study was to describe current practices on how HSSPs perform and document clinical interventions.

## 2. Materials and Methods

Survey Design: This exploratory survey was conceptualized by the Vizient Ambulatory Pharmacy Development Committee—Outcomes and Benchmarking Workgroup, which is composed of pharmacists, pharmacy administrators, and pharmacy residents from multiple health systems across the nation. Survey development followed an iterative design process. At an initial meeting, study facilitators discussed the state of practice regarding specialty pharmacy clinical interventions. During this discussion, facilitators identified several themes and areas of interest for the survey, including the definition of a clinical intervention; which healthcare professionals can perform interventions; intervention documentation practices; and utilization, analysis, and sharing practices of collected clinical intervention data.

Facilitators created an initial survey draft and presented it to the workgroup at a subsequent meeting to gather additional feedback. After incorporating revisions, workgroup members were given a final review period of 14 days to provide further feedback via email. Upon the conclusion of the review period, the survey was finalized and distributed for responses. The final survey instrument is provided as Supplementary Material Survey S1. As the survey was organizational in nature, this project was not considered human subjects research and subsequently exempt from review by an institutional review board.

The survey evaluated the types of tasks considered to be clinical intervention, non-clinical interventions, or neither within five domains (medication management, adverse drug events/monitoring, education, coordination of care, health maintenance/social determinants of health). The term “clinical intervention” was not defined within the survey, as a goal of the survey was to better understand what the respondents determined to be a clinical intervention. Task intervention type categorization was mutually exclusive, so only one option could be selected. In addition, whether or not the task was routinely documented in the organization’s practice was collected as a binary yes or no response.

The survey also evaluated which pharmacy team members could perform interventions; where and how they were documented; data elements included in documentation; and how intervention data were classified, used, reviewed, and shared with internal or external stakeholders. These documentation practices were not mutually exclusive, so multiple options could be selected for each answer.

Survey Distribution and Response Collection: The survey instrument was developed utilizing Qualtrics XM 2.70.1 (Provo, UT, USA) survey building and distribution tools. The survey was distributed for responses via two major organizational networks, the Vizient Pharmacy Network and the Vanderbilt HSSP Outcomes Research Consortium. The Vizient Pharmacy Network is a paid member-based organization composed of approximately 5000 pharmacy members from over 1400 hospitals. The Vanderbilt HSSP Outcomes Research Consortium is open to any HSSP in the United States, with no membership or fee requirements. At the time of the survey, there were approximately 48 HSSP members in the Consortium. A link to the survey was provided via email to members of the Vanderbilt HSSP Outcomes Research Consortium; the survey was distributed via a member group forum to the Vizient Pharmacy Network. The survey was open for responses from 13 April 2023 to 26 May 2023. Survey instructions directed respondents to limit responses to one per organization; however, respondents were given the option to respond anonymously.

Survey Analysis: Survey results were extracted from Qualtrics and summarized descriptively. Several questions allowed for multiple selections, and reported total percentages may exceed 100%. Data visualizations were created in Tableau 2023.2 (Seattle, WA, USA) and R 4.4.2, packages ggplot2 3.5.1 and ggpubr 0.6.0.

## 3. Results

**Figure 1:**
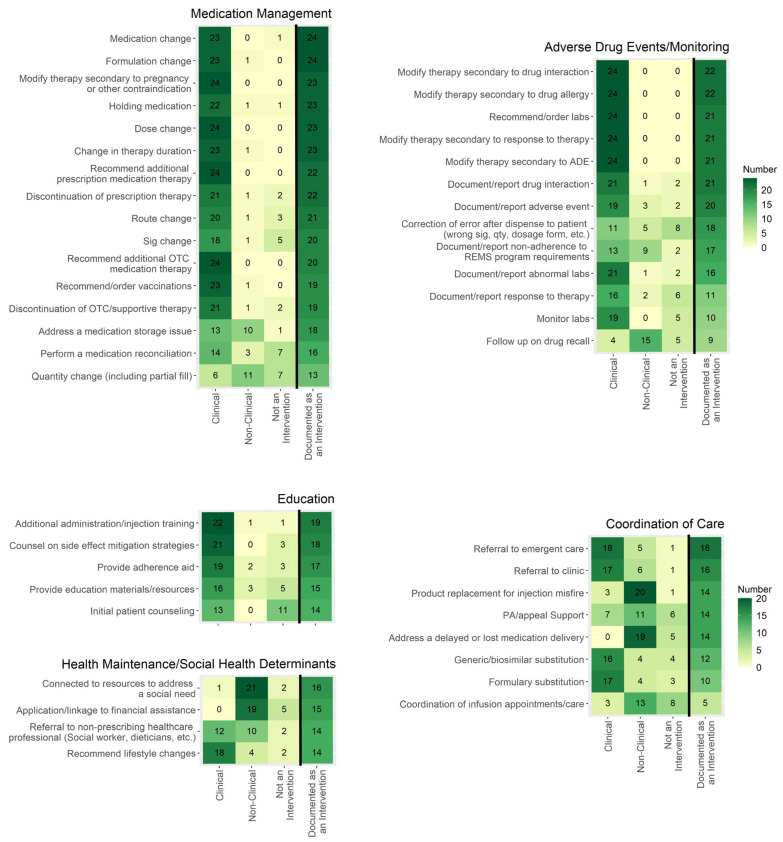
The frequency at which respondents considered tasks to be a clinical intervention, a non-clinical intervention, or not an intervention is shown in the first three columns, which are not mutually exclusive. The far-right column is mutually exclusive and shows how often respondents document these tasks as an intervention in current practice. Cells with a darker color indicate a higher frequency. Sample size *n* = 24. ADE = adverse drug event; OTC = over the counter; REMS = risk evaluation and mitigation strategy.

Twenty-four institutions responded to the survey. Figure 1 depicts various tasks addressed in the survey and whether they were determined to be a clinical intervention, a non-clinical intervention, or not an intervention by the respondents. These figures also provide the frequency at which respondents document each task as an intervention in current practice. Common themes for what HSSPs consider to be clinical and non-clinical interventions are shown in Table 1. For clinical interventions, pharmacists (100%) or pharmacy interns (38%) were performing interventions, and for non-clinical interventions, pharmacists (100%), technicians (96%), and interns (75%) performed interventions (Table 2). Other staff responsible for documenting interventions varied and included financial counselors, utilization nurses, enrollment coordinators, non-licensed office coordinators, and others.

**Figure 2:**
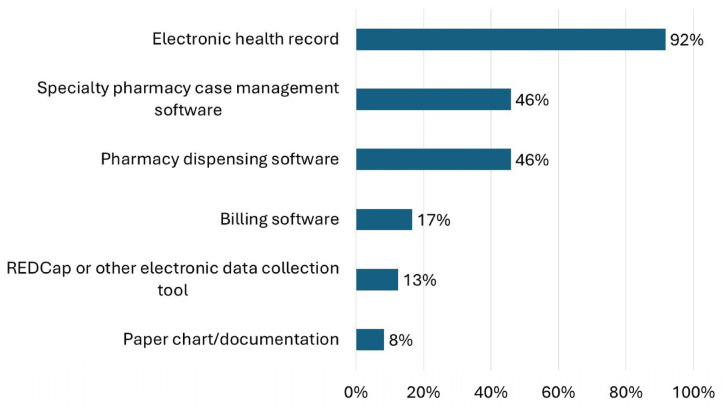
Responses to the question “Where are interventions documented?” are shown.

Interventions were most commonly documented in the EHR (92%, *n* = 22), pharmacy dispensing software (46%, *n* = 11), or specialty pharmacy case management software (46%, *n* = 11) (Figure 2). Seventeen percent of respondents (*n* = 4) reported documenting interventions in other places and described documenting in customizable EHR modules such as Epic Flowsheets or Navigators, manual Excel documentation, and other billing software. For respondents that reported documenting in the EHR, 79% (*n* = 19) documented within a specialty pharmacy-specific note or encounter, 42% (*n* = 10) within a reportable issue tracking functionality (such as “iVents” in Epic), 38% (*n* = 9) in a message to provider, 33% (*n* = 8) in a general note or encounter, and 17% (*n* = 4) in association with the medication record.

Intervention documentation elements (Table 2) included time spent performing the intervention (58%, *n* = 14), potential adverse event had intervention not been performed (42%, *n* = 10), cost or financial outcome if intervention was not performed (17%, *n* = 4), and the probability of an adverse event happening if the intervention had not been performed (8%, *n* = 2). Notably, no respondents reported documenting whether the intervention would have been performed regardless of pharmacist involvement.

**Figure 3:**
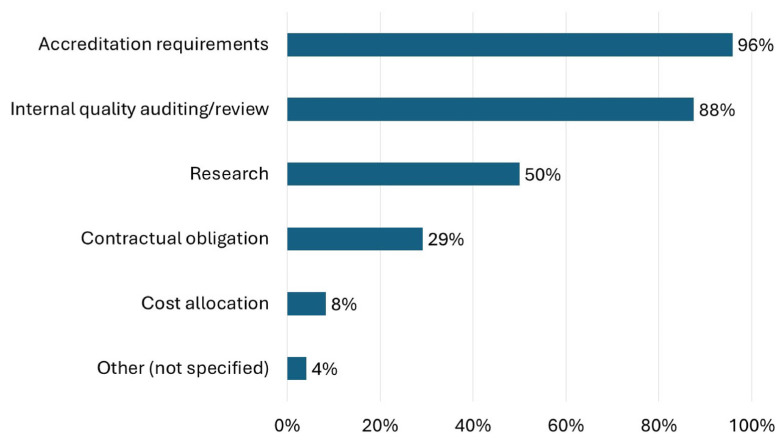
Responses to the question “Why does your organization collect intervention data?” are shown.

Intervention data were collected for the following reasons (Figure 3): accreditation requirements (96%, *n* = 23), internal quality auditing and review (88%, *n* = 21), research (50%, *n* = 12), contractual obligations (29%, *n* = 7), and cost allocation (8%, *n* = 2). Three-quarters of the respondents (75%) reported reviewing intervention data quarterly (Table 3). Internally, specialty pharmacies commonly reported intervention data to coordinators and managers (92%, *n* = 22), staff (75%, *n* = 18), and administrators (67%, *n* = 16) (Table 3). Less than half (42%, *n* = 10) of respondents stated they report intervention data to stakeholders external to the specialty pharmacy. Notably, no respondents reported sharing intervention data with patients.

Intervention data were categorized for reporting in various ways, most commonly by intervention type (88%, *n* = 21), medication (50%, *n* = 12), diagnosis or therapeutic group (42%, *n* = 10), team or individual performing the intervention (25%, *n* = 6), medication type (e.g., specialty vs. specialty-lite) (Table 3). Only 17% (*n* = 4) of respondents reported quantifying interventions’ clinical or financial value. Some strategies shared from sites that quantified the clinical or financial value of interventions included applying clinical or financial data from published literature to the interventions and calculating cost avoidance from medications that were held or stopped that otherwise would have been filled or calculating costs from prevented therapy changes, readmissions or urgent care visits.

## 4. Discussion

Results from this survey provide valuable insight into the definition, documentation, use, and reporting of HSSP pharmacist interventions. The growing body of research performed by HSSPs has demonstrated the benefits of the specialty pharmacy model [12]. Most of the current literature reporting specialty pharmacy interventions align with actions that survey respondents consider to be clinical or non-clinical interventions [6,7,8,15,16]. However, there were many tasks in the survey considered to be interventions that HSSPs have not reported, and they represent an opportunity for specialty pharmacies to demonstrate their impact. Defining and increasing the documentation and reporting of interventions can further demonstrate the value of this model by more distinctly tying pharmacist interventions to patient outcomes and decreased healthcare costs. Survey results presented herein can serve as a guide for HSSPs to improve intervention documentation practices and reporting.

### 4.1. Interventions Defined and Documented

This survey revealed common themes for what HSSPs consider to be clinical and non-clinical interventions. Of the potential interventions surveyed, most were considered to be clinical or non-clinical interventions (rather than not an intervention). However, a few categories were less likely to be considered interventions, such as medication access support, coordinating appointments, and correcting errors after dispensing (Figure 1). Medication access support is a key aspect of specialty pharmacies, as most specialty medications require a lengthy process of obtaining insurance approval and financial assistance (if needed). Several studies have demonstrated specialty pharmacy staff’s role in reducing patient out-of-pocket costs using institutional grants or connecting patients with manufacturer copay assistance, manufacturer patient assistance programs, or charitable foundation grants [16,17,18,19,20,21]. Additionally, specialty pharmacy staff help reduce turnaround time from prescription ordering to patients being approved for therapy and report high insurance approval rates [21,22,23,24,25,26,27,28,29]. Though ensuring medication access is a key role of specialty pharmacy staff, the current survey results indicate that specialty pharmacies do not deem these actions to be considered an intervention.

Conversely, tasks where a change in therapy was made or recommended, such as a medication, dose, or formulation change, were most frequently considered clinical interventions and likely to be documented as an intervention. An ASHP recent survey of HSSPs showed that pharmacists are heavily involved in specialty medication selection, sometimes using a collaborative pharmacy practice agreement to prescribe therapy [12]. Additionally, HSSPs reported completing several tasks that result in therapy changes, including discontinuing/recommending discontinuation of a medication and ordering/recommending ancillary drugs, dose adjustments, or new therapies [12]. The current survey data support that HSSPs consider these types of tasks to be interventions, and the high rate of documentation of these tasks aligns with the ASHP HSSP Clinical Services survey [12].

Tasks involving routine documentation or monitoring without action being taken were more likely to be categorized as not an intervention. Almost half of the respondents did not consider initial patient counseling to be an intervention, but when additional education was provided for issues such as administration technique or adherence, these tasks were more commonly agreed to be a clinical intervention and documented. Based on the variability of responses, no consensus has been reached on whether routine documentation or monitoring tasks that do not require additional actions should be considered an intervention. Consensus on these tasks as an intervention type would be useful, as monitoring is a very common task and could therefore skew intervention numbers if it is not consistently reported as an intervention across specialty pharmacies. Regardless of whether they are classified as interventions, documenting routine counseling and monitoring tasks in the EHR should be prioritized to demonstrate the care provided by pharmacists, enable calculation of intervention rates if deemed interventions, and support appropriate pharmacy resource allocation.

Documentation of care coordination tasks as an intervention was variable, with most categories being documented as an intervention by around 50% of respondents. Coordination of care tasks was mixed on categorization as clinical or non-clinical, with referral to additional care and therapy changes (i.e., generic or formulary substitution) more frequently considered clinical and all others non-clinical or not an intervention. One descriptive report highlights the importance of HSSPs in managing the many types of care transitions that specialty patients experience, including transitions in sites of care, between different provider types, among prescribed specialty medications, and during financial coverage changes [30]. Similar to the variability in considering documentation and monitoring an intervention, the high frequency at which HSSPs perform care coordination tasks reinforces the need for consensus on whether these tasks should be labeled and reported as interventions.

### 4.2. Intervention Documentation

The EHR was the primary tool for documenting interventions, performing pre-treatment screening of medication appropriateness, and communicating with patients, consistent with the ASHP HSSP Clinical Services Survey results [11]. Taken together, survey results indicate that HSSPs are highly advanced and invested in EHR capabilities for providing patient care and creating a transparent platform for providers, pharmacists, and patients to communicate and share medication-related information. Furthermore, research is emerging demonstrating how HSSPs are using the EHR in innovative ways to provide seamless medication management and advanced patient monitoring services [31,32].

Though over half of respondents tracked time spent on interventions, fewer documented potential adverse outcomes or financial impact if the intervention had not occurred. HSSPs may be less inclined to document potential adverse outcomes or financial information if the primary driver for documenting interventions is reporting to accrediting bodies or auditing, as was most commonly cited (Figure 3). However, quantifying the financial value of a pharmacist intervention is critical, especially in scenarios where many patients cannot use the specialty pharmacy due to payer or manufacturer restrictions, because these services are not reimbursed or covered in any way. Survey respondents reported using previous literature for applying financial value to pharmacists’ interventions. Past studies estimate pharmacist interventions save between $156,000 and $1.5 million over a few months in various settings (multiple sclerosis, pediatric ambulatory care, hematology/oncology) [6,7,8]. Estimating indirect cost avoidance from pharmacist interventions can be complex. Patanwala et al. recommend using a panel to evaluate the probability of the intervention occurring without the pharmacist (probability of trajectory change), consequences if the intervention had not occurred, and a range of probabilities for the consequences [9]. This process may not be conducive to rapid reporting or prospective documentation as part of routine care. Quantifying direct cost avoidance by pharmacist interventions resulting in medication discontinuation or changes may be more feasible. A standardized framework for documenting cost avoidance values for pharmacist intervention could improve adoption and consistency in reporting by HSSPs.

### 4.3. Implementation Considerations

Without consensus on what is considered to be an intervention and standardized reporting elements, the variability seen in the current survey results is likely to persist. Resources exist for better aligning intervention documentation. Recently, the National Association of Specialty Pharmacy (NASP) Outcomes Committee clinical intervention group developed a framework for specialty pharmacist interventions, which defined an intervention as “a clinical intervention occurs when a potential medication, therapy or healthcare issue is identified by a pharmacy team member, and additional actions are taken by a clinician to resolve the issue and positively impact patient care [33].” The group recommended that interventions related to six categories be documented: safety, medicine optimization, adherence/persistence, affordability, supply chain impact, and health and coordination of care. Elements to be documented within interventions and metrics that could be tracked related to interventions, including intervention success rate, likelihood of adverse events, and cost efficiency, were recommended [33]. The framework was based on the Pharmacy Quality Alliance’s (PQA) medication therapy problem categories, which standardizes how identified problems are categorized [34]. Broad dissemination and adoption of the NASP suggested intervention framework could improve uptake and implementation, potentially leading to improvements in standardized intervention reporting and benchmarking.

The current survey also identified areas for improvement in communicating interventions. Though 90% of respondents reported interventions to accrediting bodies, only 60% reported interventions to hospital/health-system administration, and none reported them to patients. This finding somewhat aligns with the ASHP HSSP Clinical Services Survey data in that very few HSSPs communicated specialty pharmacy metrics to patients, but most reported metrics to hospital/health-system administration [11]. Patients often do not have a choice in which specialty pharmacy they can use for dispensing their specialty medication due to frequent manufacturer-limited distribution networks and payer network restrictions. However, patients should be considered important stakeholders in the specialty pharmacy field and could benefit from being better informed about pharmacy metrics and interventions performed by the pharmacy through which they are receiving medication.

Limitations: This study was limited by a small sample size and the potential for duplicates, as sites could have responded anonymously. A response rate could not be determined since the survey was disseminated via a large community network post. Survey results likely do not incorporate all HSSP perspectives and may therefore be limited in generalizability, but similar results were seen in larger surveys conducted of this population where questions overlapped [11]. Additionally, responding sites’ demographic data were not collected, so no comparisons between types of HSSP models, sizes, geographic location, or other characteristics were performed, which could provide further insight into documentation variability.

## 5. Conclusions

HSSP pharmacists perform several interventions to ensure patients are prescribed and able to access appropriate therapy and benefit from treatment once prescribed. Documenting and reporting pharmacist interventions are essential to demonstrate pharmacists’ role and value in specialty pharmacy. The national survey study results presented here provide useful insight into what HSSPs consider to be a pharmacist intervention, how and where interventions are documented, and how this data is used. Respondents commonly consider clinical interventions to be actions that impact a patient’s treatment plan, including therapy adjustments, patient education beyond initial education, and referrals for further medical evaluation. Based on the survey results, the authors support the recently proposed NASP definition of a clinical intervention (“a clinical intervention occurs when a potential medication, therapy or healthcare issue is identified by a pharmacy team member, and additional actions are taken by a clinician to resolve the issue and positively impact patient care”) [33]. However, it would be beneficial to further define what constitutes “additional actions” as interventions. The current survey suggests that routine education and monitoring activities, resolving prescription issues (e.g., access, prescription error, or delivery), connecting patients to social/financial support resources, and optimizing dispense quantities to reduce waste should be considered non-clinical interventions. The authors agree that all tasks should be documented to help HSSPs collect workload metrics and demonstrate pharmacists’ value. A standardized framework, such as the one proposed by NASP for specialty pharmacist interventions, developed and agreed upon by multiple specialty pharmacy stakeholders, could improve implementation and benchmarking.

## Figures and Tables

**Table 1:** Themes for intervention categorization.

Clinical Intervention	Performing a task that results in an alteration or reevaluation of patients’ therapeutic course Educating patients on therapy management or issues that arise throughout the course of treatment Referring patients to healthcare providers for evaluation including clinic and urgent care
Non-clinical intervention	Addressing medication availability, recall, replacement, and delivery issues Connecting patients to resources to address social and financial needs Adjusting medication dispense quantities for waste avoidance efforts
Less likely to be considered an intervention	Correcting prescription errors post-dispensing Coordinating care for infusion appointments Initial patient counseling

**Table 2:** Intervention details.

Question	Response	Number (*n* = 24)	Percentage
Who can perform clinical interventions at your organization?	Pharmacists	24	100.0%
	Interns	9	37.5%
	Technicians	3	12.5%
Who can perform non-clinical interventions at your organization?	Pharmacists	24	100.0%
	Technicians	23	95.8%
	Interns	18	75.0%
	Other (enrollment coordinators, financial counselors, office coordinator, nurses, students)	6	25.0%
How are interventions documented in the electronic health record?	Specialty pharmacy-specific note/encounter documentation	19	79.2%
	Reportable issue tracking functionality (e.g., iVent/MTP)	10	41.7%
	Message to provider	9	37.5%
	General note/encounter documentation	8	33.3%
	Association with the medication record	4	16.7%
	Other (Epic Flowsheets and Specialty Navigator, Excel Document)	1	4.2%
Indicate if these elements are included in your organization’s intervention documentation	Cost/financial outcome if intervention was not performed	4	16.7%
	Time spent performing the intervention	14	58.3%
	Potential adverse event had intervention not been performed	10	41.7%
	Probability of adverse event happening if intervention had not been performed	2	8.3%
	Would the intervention have been made regardless of pharmacist involvement	0	0.0%

**Table 3:** Use of intervention data.

Question	Response	Number (*n* = 24)	Percentage
How often does your organization review intervention data?	Quarterly	18	75.0%
	Monthly	7	29.2%
	As needed	7	29.2%
	Daily	3	12.5%
	Yearly	3	12.5%
	Unscheduled	1	4.2%
	Weekly	0	0.0%
Which internal stakeholders receive reports of intervention data?	Coordinators/Managers	22	91.7%
	Staff	18	75.0%
	Administrators	16	66.7%
	None	0	0.0%
Does your organization report data to external stakeholders?	No	14	58.3%
	Yes	10	41.7%
Which external stakeholders receive reports of intervention data? (*n* = 10)	Accrediting bodies	9	90.0%
	Clinic providers	7	70.0%
	Payors	7	70.0%
	Hospital/health-system administration	6	60.0%
	Other (manufacturers, REMS programs)	1	10.0%
	Patients	0	0.0%
How does your organization categorize intervention data in reporting?	Type of intervention	21	87.5%
	Medication	12	50.0%
	Diagnosis or therapeutic group	10	41.7%
	Team/individual performing the intervention	6	25.0%
	Medication type (i.e., specialty vs. specialty-lite)	5	20.8%
	Adverse outcome avoided by from intervention	2	8.3%
Does your organization quantify the clinical or financial value of interventions?	No	20	83.3
	Yes	4	16.7%

## Data Availability

All data generated or analyzed during this study are included in this published article.

## Supplementary Materials

The following supporting information can be downloaded at: https://www.mdpi.com/article/10.3390/pharmacy13060172/s1, Survey S1: Vizient Outcomes and Benchmarking Workgroup | Specialty Pharmacy Clinical Intervention Survey.

## Abbreviations

The following abbreviations are used in this manuscript:

HSSP	Health-system specialty pharmacy
EHR	Electronic health record
ASHP	American Society of Health System Pharmacists
ACHC	Accreditation for Healthcare Commission
TJC	The Joint Commission
PQA	Pharmacy Quality Alliance
NASP	National Association of Specialty Pharmacy
