# Supplementary material for: Capturing Value: How Health-System Specialty Pharmacies Define and Document Pharmacist Interventions
Source: Pharmacy (Basel). 2025 Nov 26;13(6):172. doi: 10.3390/pharmacy13060172 (PMC12736702; doi:10.3390/pharmacy13060172)
Supplement: Supplementary file 1 [file pharmacy-13-00172-s001.zip › pharmacy-3935839-supplementary.pdf]

Vizient Outcomes and Benchmarking Workgroup | Specialty Pharmacy Clinical Intervention Survey

Introduction

This survey is intended to examine specialty pharmacy practices for identifying, documenting, and reporting on clinical and non-clinical interventions performed by pharmacy staff. Thank you for your participation! If possible, please limit responses to a single response per organization, however multiple persons within an organization may contribute to that single response.

Provide the name of the responding organization (optional)

Definition of an Intervention

Which of the following do you consider as clinical or non-clinical interventions?

	Clinical	Non-Clinical	Not an Intervention
**Adverse Drug Events/Monitoring**			
Correction of error after dispense to patient (wrong sig, qty, dosage form, etc.)	○	○	○
Document/report adverse event	○	○	○
Document/report drug interaction	○	○	○
Document/report response to therapy	○	○	○
Follow up on drug recall	○	○	○
Modify therapy secondary to ADE	○	○	○
Modify therapy secondary to drug interaction	○	○	○
Modify therapy secondary to response to therapy	○	○	○
Monitor labs	○	○	○
Recommend/order labs	○	○	○
**Coordination of Care**			
Coordination of infusion appointments/care	○	○	○

	Clinical	Non-Clinical	Not an Intervention
Formulary substitution	○	○	○
Generic/biosimilar substitution	○	○	○
Referral to clinic	○	○	○
Referral to emergent care	○	○	○
PA/appeal Support	○	○	○

Education

	Clinical	Non-Clinical	Not an Intervention
Additional administration/injection training	○	○	○
Patient counseling	○	○	○
Provide adherence aid	○	○	○
Provide education materials/resources	○	○	○

Health Maintenance / Social Determinants of Health

	Clinical	Non-Clinical	Not an Intervention
Application/linkage to financial assistance	○	○	○
Connected to resources to address a social need	○	○	○
Recommend lifestyle changes	○	○	○

	Clinical	Non-Clinical	Not an Intervention
Referral to non-prescribing healthcare professional (Social worker, dieticians, etc.)	○	○	○

Medication Management

	Clinical	Non-Clinical	Not an Intervention
Change in therapy duration	○	○	○
Discontinuation of therapy	○	○	○
Dose change	○	○	○
Formulation change	○	○	○
Holding medication	○	○	○
Medication change	○	○	○
Recommend additional OTC medication therapy	○	○	○
Recommend additional prescription medication therapy	○	○	○
Quantity change (including partial fill)	○	○	○
Route change	○	○	○
Sig change	○	○	○

Which of the following actions does your organization require to be specifically documented as an intervention?

i.e. action is identified as an intervention by your organization, and requires additional documentation beyond that of the normal workflow when performed

	Yes	No
**Adverse Drug Events/Monitoring**		
Correction of error after dispense to patient (wrong sig, qty, dosage form, etc.)	○	○
Document/report adverse event	○	○
Document/report drug interaction	○	○
Document/report response to therapy	○	○
Follow up on drug recall	○	○
Modify therapy secondary to ADE	○	○
Modify therapy secondary to drug interaction	○	○
Modify therapy secondary to response to therapy	○	○
Monitor labs	○	○
Recommend/order labs	○	○
**Coordination of Care**		
Coordination of infusion appointments/care	○	○
Formulary substitution	○	○

	Yes	No
Generic/biosimilar substitution	○	○
Referral to clinic	○	○
Referral to emergent care	○	○
PA/appeal Support	○	○

Education

Additional administration/injection training	○	○
Patient counseling	○	○
Provide adherence aid	○	○
Provide education materials/resources	○	○

Health Maintenance / Social Determinants of Health

Application/linkage to financial assistance	○	○
Connected to resources to address a social need	○	○
Recommend lifestyle changes	○	○
Referral to non-prescribing healthcare professional (Social worker, dieticians, etc.)	○	○

	Yes	No
**Medication Management**		
Change in therapy duration	○	○
Discontinuation of therapy	○	○
Dose change	○	○
Formulation change	○	○
Holding medication	○	○
Medication change	○	○
Recommend additional OTC medication therapy	○	○
Recommend additional prescription medication therapy	○	○
Quantity change (including partial fill)	○	○
Route change	○	○
Sig change	○	○

Are there any actions your organization would consider an intervention that are missing from this list?

- ○ Yes
- ○ No

Please describe any missing clinical/nonclinical interventions

[ ]

Performing/Documenting Interventions

Who can perform clinical interventions at your organization?

- [ ] Pharmacists
- [ ] Technicians
- [ ] Pharmacist interns
- [ ] Other

Please describe other staff performing clinical interventions

[ ]

Who can perform nonclinical interventions at your organization?

- ☐ Pharmacists
- ☐ Technicians
- ☐ Pharmacist interns
- ☐ Other

Please describe other staff performing nonclinical interventions

Where are interventions documented?

- ☐ Electronic health record
- ☐ Pharmacy dispensing software
- ☐ Specialty pharmacy case management software
- ☐ REDCap or other electronic data collection tool
- ☐ Paper chart/documentation
- ☐ Other

Please describe other places where interventions are documented

How are interventions documented in the EHR?

- [ ] General note/encounter documentation
- [ ] Specialty pharmacy-specific note/encounter documentation
- [ ] Association with the medication record
- [ ] Reportable issue tracking functionality (e.g. iVent/MTP)
- [ ] Message to provider
- [ ] Other

Please list other places documented in the EHR

Indicate if these elements are included in your organization's intervention documentation

- [ ] Time spent performing the intervention
- [ ] Potential adverse event had intervention not been performed
- [ ] Probability of adverse event happening if intervention had not been performed

- [ ] Would the intervention have been made regardless of pharmacist involvement
- [ ] Cost/financial outcome if intervention was not performed

Utilization of Intervention Data

Why does your organization collect intervention data?

- [ ] Accreditation requirements
- [ ] Cost allocation
- [ ] Contractual obligation
- [ ] Internal quality auditing/review
- [ ] Research
- [ ] Other

What other reasons does your organization collect intervention data?

How often does your organization review intervention data?

- [ ] Daily

- [ ] Weekly
- [ ] Monthly
- [ ] Quarterly
- [ ] Yearly
- [ ] As needed
- [ ] Unscheduled

To which internal stakeholders does your organization report intervention data?

- [ ] Administrators
- [ ] Coordinators/Managers
- [ ] Staff
- [ ] None

Does your organization report intervention data to other stakeholders outside of the specialty pharmacy?

- ○ Yes
- ○ No

Which outside stakeholders receive reports of intervention data?

- ☐ Accrediting bodies
- ☐ Clinic providers
- ☐ Hospital/health system administration
- ☐ Patients
- ☐ Payors
- ☐ Other

Which other outside stakeholders receive reports of intervention data?

How does your organization classify intervention data in reporting?

- ☐ By type of intervention
- ☐ By medication
- ☐ By diagnosis or therapeutic group
- ☐ By team/individual performing the intervention
- ☐ By medication type (i.e. specialty vs. specialty-lite, rare/orphan, etc.)
- ☐ By adverse outcome avoided by from intervention

Does your organization quantify the clinical or financial value of interventions?

- ○ Yes
- ○ No

Briefly describe how your organization quantifies value of interventions

Powered by Qualtrics
